# Effects of Oscillatory Flow on Fertilization in the Green Sea Urchin *Strongylocentrotus droebachiensis*

**DOI:** 10.1371/journal.pone.0076082

**Published:** 2013-09-30

**Authors:** Louise T. Kregting, Anna L. Bass, Òscar Guadayol, Philip O. Yund, Florence I. M. Thomas

**Affiliations:** 1 Marine Science Center, University of New England, Biddeford, Maine, United States of America; 2 Hawai’i Institute of Marine Biology, University of Hawai’i, Kane’ohe, Hawai’i, United States of America; Universidad Nacional Autónoma de México, Mexico

## Abstract

Broadcast spawning invertebrates that live in shallow, high-energy coastal habitats are subjected to oscillatory water motion that creates unsteady flow fields above the surface of animals. The frequency of the oscillatory fluctuations is driven by the wave period, which will influence the stability of local flow structures and may affect fertilization processes. Using an oscillatory water tunnel, we quantified the percentage of eggs fertilized on or near spawning green sea urchins, *Strongylocentrotus droebachiensis*. Eggs were sampled in the water column, wake eddy, substratum and aboral surface under a range of different periods (*T* = 4.5 – 12.7 s) and velocities of oscillatory flow. The root-mean-square wave velocity (rms(*u*_w_)) was a good predictor of fertilization in oscillatory flow, although the root-mean-square of total velocity (rms(*u*)), which incorporates all the components of flow (current, wave and turbulence), also provided significant predictions. The percentage of eggs fertilized varied between 50 – 85% at low flows (rms(*u*_w_) <0.02 m s^−1^), depending on the location sampled, but declined to below 10% for most locations at higher rms(*u*_w_). The water column was an important location for fertilization with a relative contribution greater than that of the aboral surface, especially at medium and high rms(*u*_w_) categories. We conclude that gametes can be successfully fertilized on or near the parent under a range of oscillatory flow conditions.

## Introduction

Many free-spawning benthic invertebrates live in energetic coastal environments where variation in water motion will exert a major influence on fertilization processes. In the simplest sense, water motion experienced by an organism is composed of underlying currents, ocean and local wind driven oscillations and turbulence. In unidirectional flow, gametes of a spawning animal are transported by the currents and turbulent diffusion, which create a spatially spreading plume of gametes downstream of the animal [1], [2]. Oscillatory flow is characterized by orbital motion. As flow approaches the seabed the vertical portion of this flow is damped creating horizontal oscillatory water motion. Thus animals spawning in oscillatory flow experience reversing horizontal flows in addition to currents and turbulent diffusion. Further, as water moves past an organism, vortices are created in the downstream wake [3]. The extent of vortex shedding depends on velocity of the flow, as well as the period of reversals. Thus the time scales of variation in water motion that affect the movement of gametes of free-spawning benthic organisms in unidirectional vs. oscillatory motion are quite different and this difference is likely to have important implications for the success of fertilization processes.

Investigations to date have focused largely on the influences of unidirectional flow on fertilization processes of benthic organisms that spawn externally [4]–[6]. In steady unidirectional flow the shear created at the surface of a spawning animal is constant and depends on flow velocity, animal size and morphology. Furthermore, the wake downstream of the animal potentially constitutes an important site for fertilization [5]. Past work [4], [5], [7] has demonstrated that as velocity increases, fertilization levels in benthic organisms decrease owing to increased advection and turbulent diffusion of gametes, which decreases concentrations of gametes to levels below that required for effective fertilization [7]–[9].

While studies based on unidirectional flow regimes have significantly increased our understanding of fertilization processes for organisms that live in environments dominated by tidal currents, many broadcast-spawning invertebrates live in high-energy shallow coastal environments where oscillatory water motion from locally generated or oceanic wind waves is the norm. In spite of the prevalence of this habitat, no quantitative studies have determined the influence of oscillatory flow on fertilization in benthic organisms (but *see* Denny et al. [10] for work in surge channels on wave-swept shores). In oscillatory flow, velocity gradients formed as water flows in one direction are quickly disrupted as the flow reverses. Consequently, the shear layers are constantly forming and shedding vortices; high shear stresses are potentially produced close to the surface of the animal, with the frequency of these perturbations determined by the magnitude of flow, structure size and period of reversals [11]. Thus it may be expected that fertilization in oscillatory flow will be dependent in part on the magnitude and period of the oscillatory wave.

The interaction between hydrodynamics and the biology of the organism is also important. Gametes are typically released in a viscous fluid that, under steady flow conditions, can form piles on the surface of a spawning animal or clumps and strings that drift into the water column, thus releasing eggs and sperm over time [12], [13]. Because velocity is reduced in the boundary layer at the surface of an organism, the presence of gamete piles can enhance fertilization by reducing the rate of diffusion of sperm from a male and providing sperm to the eggs of a downstream female over a period of several hours [14]. At a critical stage in the flow regime, however, a shear threshold will be reached where gametes are ablated away from the surface of the parent [5]. In sea urchins, the negatively buoyant eggs can be entrained in an eddy downstream of a female or fall to the substrate, with many eggs fertilized in these locations [5]. Therefore under steady unidirectional flow conditions, fertilization in sea urchins can take place at a number of identifiable locations near the parent. In an oscillatory flow regime, however, the continuously accelerating and decelerating flow over and around the organism generates vortices that are likely to have a major influence on gamete dispersion and hence the location and level of fertilization.

Here we present results from a laboratory experiment on fertilization in green sea urchins, *Strongylocentrotus droebachiensis,* subjected to different combinations of velocities and periods of oscillatory motion. Our objectives were to 1) quantify the percentage of eggs fertilized in the water column, in the wake eddies, on the substratum, and on the aboral surface of a spawning sea urchin for a range of periods and velocities relevant to local field populations, 2) determine an appropriate hydrodynamic parameter for relating the water flow to the fertilization processes, and 3) determine the relative contribution to overall fertilization rates of different spatial areas adjacent to individual animals.

## Materials and Methods

### Ethics Statement

The sea urchins were purchased from commercial sea urchin divers who hand collected them under a commercial license. The species *Strongylocentrotus droebachiensis* is not an endangered species.

### Oscillatory water tunnel

**Figure 1:**
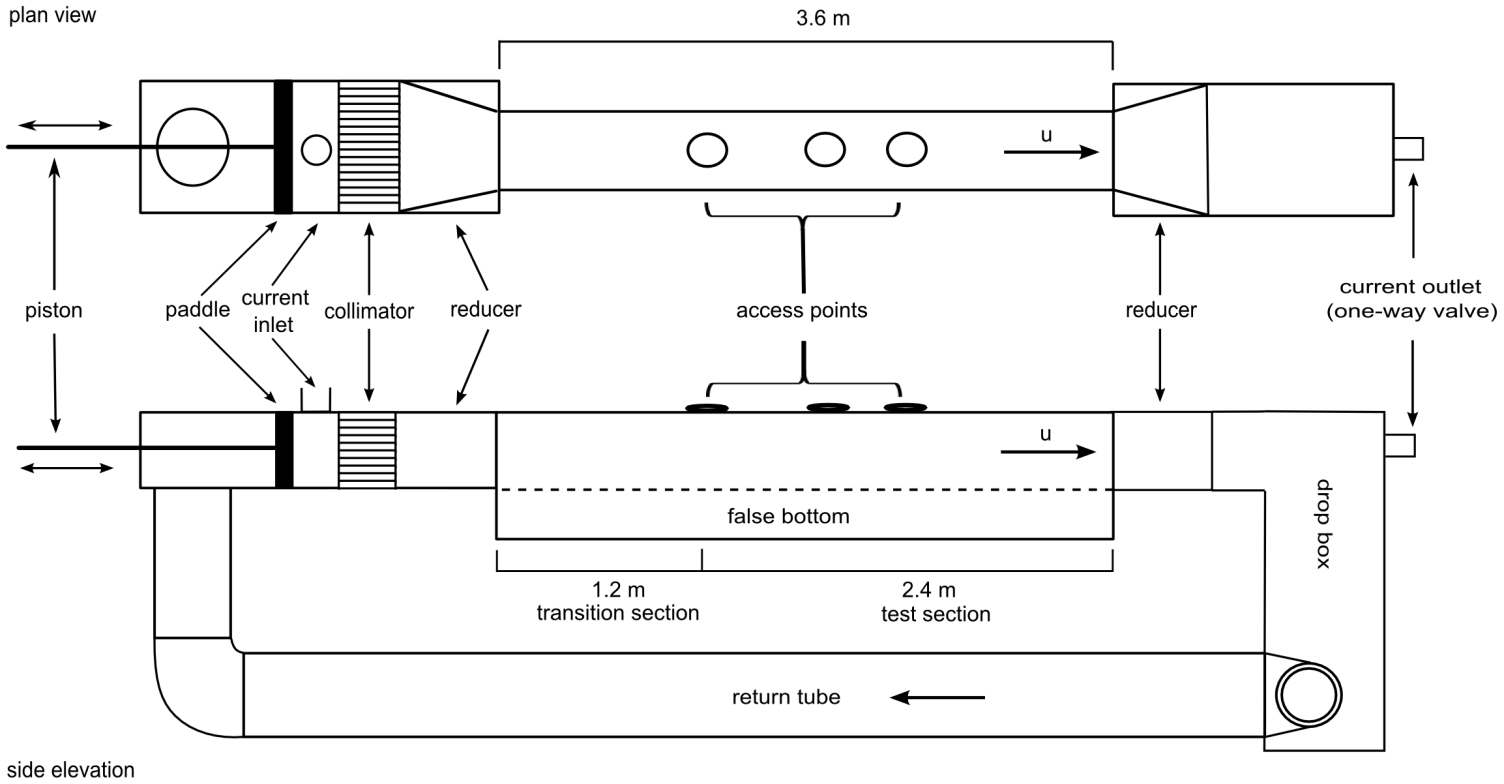
Diagram of the oscillatory water tunnel (OWT). The OWT chamber was used to determine the effects of oscillatory flow on fertilization of the green sea urchin *Strongylocentrotus droebachiensis*. The drive mechanism that attaches to the paddle (via two coupled hydraulic pistons driven by a flywheel attached to an electric motor powered by an adjustable frequency drive) has been omitted from the figure.

**Figure 2:**
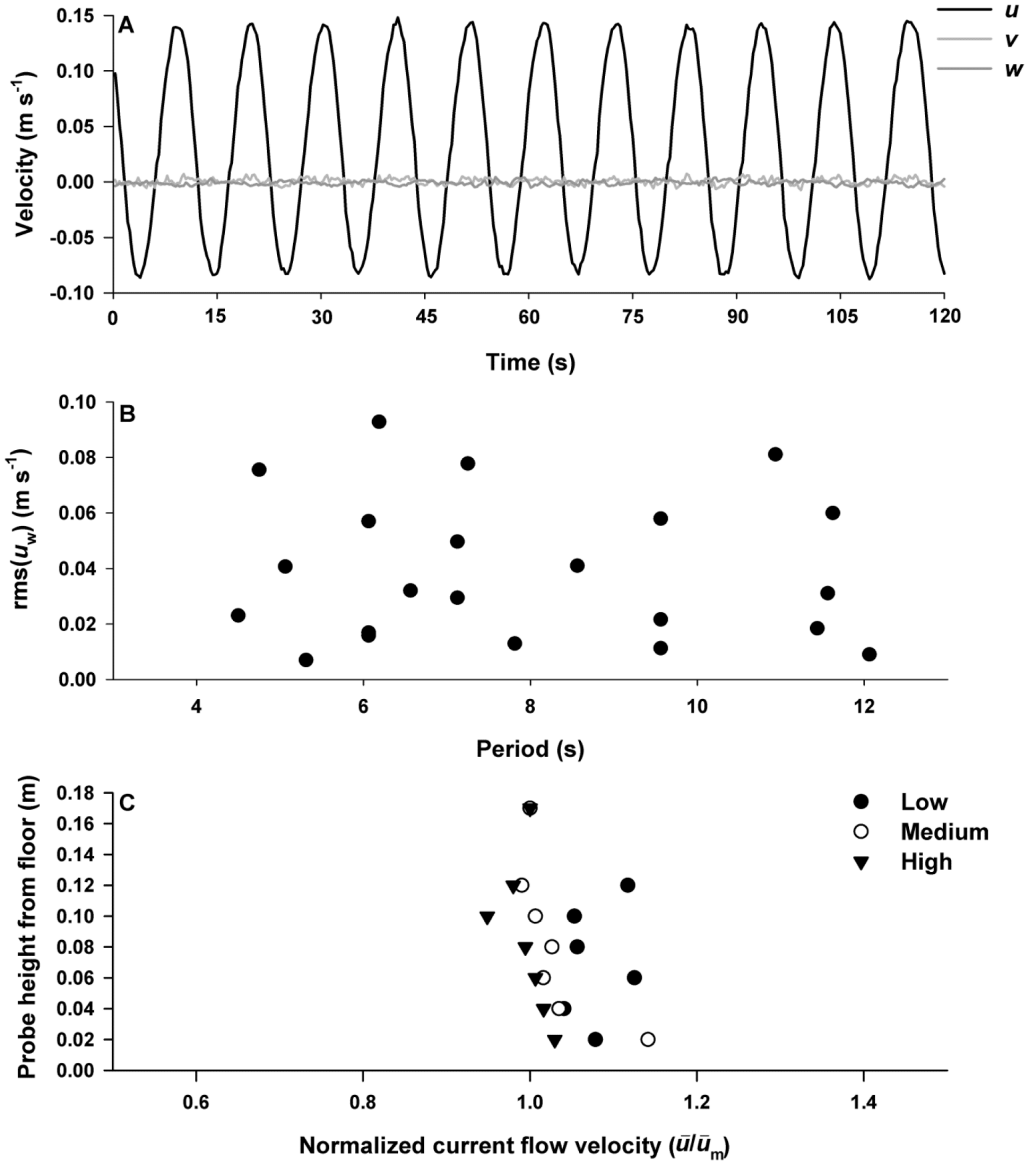
Hydrodynamic characterisation of the oscillatory water tunnel. (A) Sample wave form with underlying current (*u*, *v*, and *w* velocities (m s^−1^)) from an ADV positioned with the *x* probe oriented with the dominant current direction and the sample volume 0.04 m above the substrate (corresponding to the average height of the female sea urchins used in the trials). (B) Range of hydrodynamic conditions explored in the experimental trials, with both rms(*u*_w_) (m s^−1^) and period (s) manipulated independently. (C) Test section velocity profiles in the absence of the female sea urchin. Mean longitudinal velocity (*ū*) normalized by mean longitudinal velocity of the maximum probe height (*ū*_m_) from the tank floor (0.18 m) from three velocity profiles representative from each of the rms(*u*_w_) categories: LOW [< 0.03 m s^−1^], MEDIUM [> 0.03<0.06 m s^−1^], HIGH [> 0.06 m s^−1^]

Experiments were conducted in a fully enclosed acrylic oscillatory water tunnel (OWT) with a square working section of 3.6 length (L) ×0.31 width (W) ×0.31 height (H) m (Fig. 1) designed to mimic the oscillatory motion of breaking ocean swells. Oscillatory motion was produced by a flywheel attached to a motor, with motor rotational velocity controlled by an adjustable frequency drive (the flywheel, motor and adjustable frequency drive are not shown in Fig. 1). This oscillatory motion was transferred to the water in the tunnel via a paddle driven by two coupled hydraulic piston cylinders. The resulting wave forms were sinusoidal with essentially no vertical or transverse water motion (*see* example in Fig. 2A). The driving cylinder was attached to the flywheel at various points that were different distances from the center so that both the period and velocity could be independently manipulated to produce a range of oscillatory conditions (Fig. 2B). Because of size limitations and constrained flow, this OWT primarily reproduces reversals of flow and the development of shear layers on the urchin, but does not allow for full development of boundary conditions above the substratum, damps the vertical components of flow, and may not provide small-scale turbulence conditions that scale appropriately with wave period and velocity.

To prevent saturation of the water tunnel with gametes due to back and forth mixing, and to simulate a unidirectional current underlying the waves (e.g., a tidal current oriented parallel to waves), a flow-through current of approximately 0.03 m s^−1^ was introduced in the water tunnel. This current was evenly distributed along the vertical axis of the water column (Fig. 2C). Ambient seawater entered the OWT via a dispersing inlet pipe positioned perpendicular to the long axis of the OWT between the paddle and the collimator (Fig. 1) and exited through a one-way outlet valve at the end of the OWT. Seawater not exiting the OWT flowed into a drop-box connected to the upstream end of the OWT with a pipe to create a continuous water connection to balance the flow on both sides of the paddle. However, any remaining sperm were unable to pass the paddle and re-enter the working section. To create orderly flow through the test section, a collimator was placed at the entrance of the working section followed by a reducer (Fig. 1). Another reducer was located at the end of the working section. The floor of the OWT was covered with Lego™ base plates (0.25×0.25 m) glued to a 0.009 m thick acrylic sheet that provided a uniform rough surface as well as a substrate for securing the animals.

Sea urchins were secured in the center of the OWT located 1.24 and 2.24 m (male and female respectively) downstream from the reducer section in the test section of the OWT. Each sea urchin was secured by first holding the individual in place with rubber bands on separate small modified Lego™ plates (0.065 (L) ×0.045 (W) ×0.003 (H) m), which attached easily and quickly to the floor of the OWT with minimal disruption to the animal. Portholes on the otherwise sealed top of the working section provided access to the animals. Preliminary dye experiments were carried out to ensure that gamete concentrations were not constrained by diffusing to the sidewalls until well downstream of the sampling locations. These preliminary tests were conducted under the full range of hydrodynamic conditions used in the trials.

Water velocity measurements were obtained using an Acoustic Doppler Velocimeter (SonTek ADVField) to characterize flow at the height of the urchin when the urchin was not present. After each trial the sea urchins were removed from the OWT and the ADV was positioned vertically above where the female had been secured, with the sample volume 0.04 m above the substrate (corresponding to the average height of the female sea urchins used in the trials: 0.037 m ± 0.001 SE). The ADV sample volume was located 5 cm from the probe head and occupied <0.1 cm^3^. The vertical position of the probe minimized both the area perpendicular to any vertical flow and the volume of instrument submersed, thus minimizing the effect of any vortices shed from the instrument. The very low vertical velocities observed in all cases ensure that any small vortices shed from the probe head did not significantly affect the sampling volume. The *x* sensor was oriented with the dominant current direction. After the underlying current and oscillatory motion were established at the trial hydrodynamic condition for 1 minute, velocity was recorded for 2 minutes at a sampling frequency of 16 Hz. Velocimeter signal-to-noise ratios (SNR) were consistently above 20 dB and the correlation values for the 3 sensors remained above 90%. Before flow parameters were calculated, spikes were removed following 3D phase space threshold techniques [15], [16]. Values with correlations <60 and SNR <20 were also removed [17].

Previous fertilization experiments based on steady flow conditions have primarily used the mean flow (*ū*) to determine the sensitivity of fertilization to velocity [5], [18], [19], which in predominantly unidirectional flow is a measure of the flow magnitude and is composed of both the current and turbulence components. Because we were interested in understanding the effect of the various components of oscillatory flow on fertilization, the flow signal was decomposed. In oscillatory flow, a measurement of water velocity contains the current, the magnitude of wave driven flow, the turbulence, and instrumental noise. These components can be distinguished by extending Reynolds decomposition of the signal so that the total flow signal in the predominant direction (*u*) may be decomposed as:
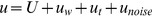
(1)


where *U* represents the low frequency component of the signal (that is, the underlying current), *u_w_* is the oscillatory component (wave component), *u_t_* is the turbulent component and *u_noise_* represents the instrumental white noise. Squaring and averaging the terms yields:
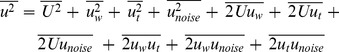
(2)


Assuming that all components are uncorrelated among themselves (that is, that all double products are 0), and that 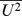  =  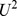 the equations simplifies to: 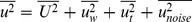
(3)


**Figure 3:**
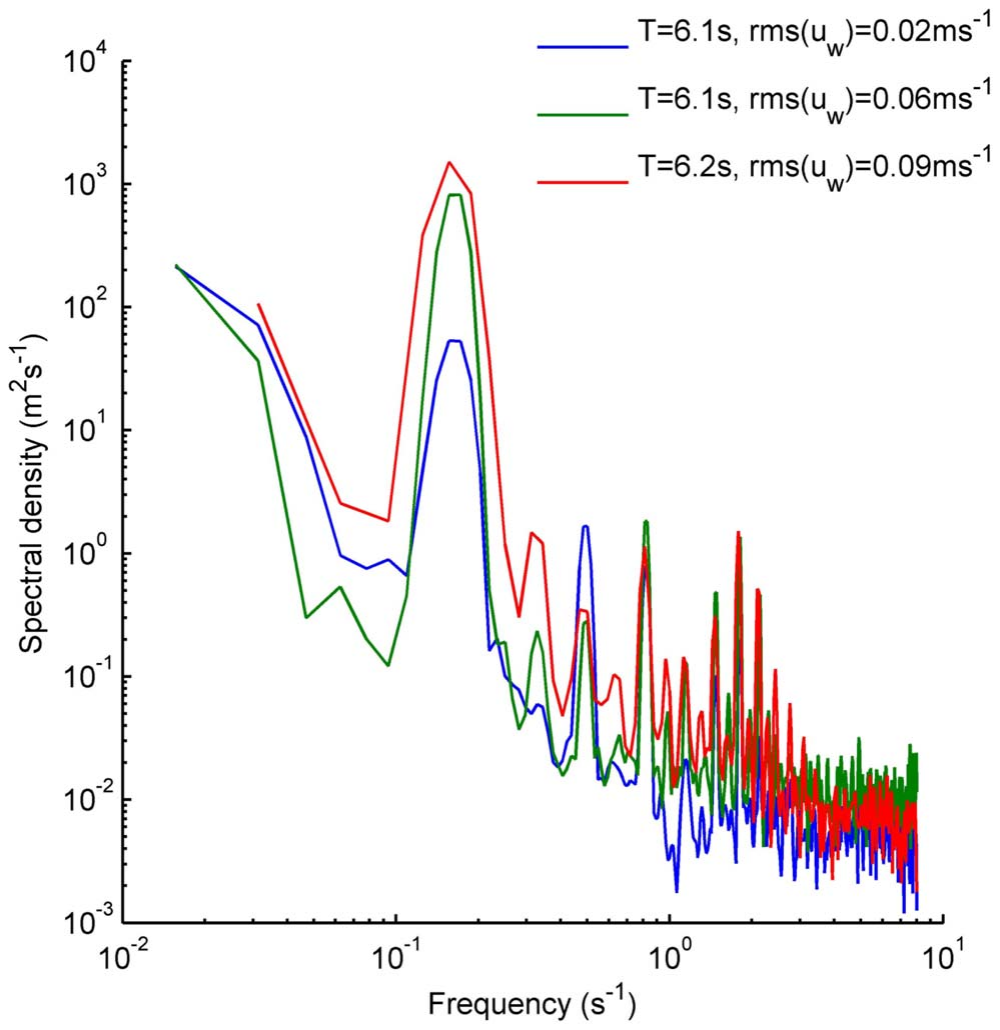
Example spectral density plots. Spectral density plots calculated from vertical velocity components representative from each of the rms(*u*_w_) categories: LOW [< 0.03 m s^−1^, blue], MEDIUM [> 0.03<0.06 m s^−1^, green], HIGH [> 0.06 m s^−1^, red]

The square roots of the elements in this equation are actually the root-mean-square (rms) velocities of the different components. It is possible to estimate these components directly from the energy density spectrum (Fig. 3) [20], since the integral of the spectral density equals the variance of the signal as long as the time series is sufficiently long. This process can be repeated for flows perpendicular to the mainstream (*v*) and vertical (*w*).

For each time series, the white noise was identified as a flat segment appearing at the highest frequencies in the power density spectrum, and its variance was computed integrating this noise level over all frequencies of the spectral density. The turbulent component (rms(*u_t_*)) was obtained by the integration of the inertial subrange (i.e., the segment of the spectral density that follows a –5/3 slope), after subtraction of the white noise. The components were calculated from vertical velocities only because, due to the geometry of the probe, this component has the lowest noise level and the observed inertial subrange is wider and more easily identifiable [21]. In addition, since the oscillating motion was generated mostly along the horizontal long axis of the OWT, the vertical spectra showed little or no peak at the oscillation frequency.

The wave component (rms(*u_w_*) was not calculated from the spectral analyses because the time series were not sufficiently long to adequately resolve spectral densities at the frequencies of oscillation, particularly for experimental conditions with longer periods. Therefore, an alternative approach was followed to calculate rms(*u_w_*). For each oscillatory experiment, the period of oscillation (*T, s* ^−*1*^) was estimated as the first local maximum in the autocorrelation function. The signal was ensemble averaged using period as the fundamental length. Then rms(*u_w_*) was estimated as the root-mean-square of the resulting ensembled wave [22].

In addition, to these flow parameters, we calculated a Keulegan-Carpenter number which relates the wave orbital excursion length to the object size (*KC*; Keulegan and Carpenter [23]):
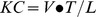
(4)


where *V* is a flow parameter, here we used rms(*u_w_*) as it was the best predictor for fertilization (*see* below) and L is the relevant length scale (sea urchin width, m). *KC* is used widely in civil and coastal engineering as a dimensionless number to describe the importance of drag forces relative to inertial forces for solid objects [24] and is related to the tendency of an object to shed vortices [11].

Fertilization experiments were conducted under a range of hydrodynamic conditions with both period (*T*  =  4.5 – 12.1 s^−1^) and rms(*u_w_*) (0.007 – 0.093 m s^−1^) manipulated independently in a total of 22 hydrodynamic trials (Fig. 2B). Flow conditions did not vary during an experiment. These conditions were within the range where dye experiments showed that gametes would not reach the walls and become constrained over the experimental distances. The periods were representative of those observed from a buoy located in Penobscot Bay (NERACOOS F01), and flows were representative of those measured in the field at the height of *S. droebachiensis* sea urchins in Pemaquid Point (rms(*u_w_*) (0.003 – 0.164 m s^−1^)) (unpublished data) on the north-east coast of Maine. *Strongylocentrotus droebachiensis* populations are exposed to a wide range of flow conditions as they are found in depths ranging from 0 – 300 m. Prior to recent harvesting, however, the bulk of the population was located below the surge zone in depths of 7–16 m [25].

### Assessing fertilization several

To assess the effect of period and flow velocity on fertilization in the green sea urchin *S. droebachiensis* at different sampling locations near a spawning female (water column, wake eddy, substrate and aboral surface), we conducted a series of experiments during the spawning season, March to April 2007. Sea urchins were hand collected from the coast of Maine and housed in flow-through seawater tanks in ambient temperatures ranging between 2 – 4.5°C. For each hydrodynamic trial (n = 22), several sea urchins with an average test width of 0.065 + 0.001 SE m were initially selected and induced to spawn by injection of ∼ 3 mL of 0.5 mol L^−1^ potassium chloride (KCl) into the coelomic cavity. Of these urchins induced to spawn, only one female and one male animal that were spawning from at least 4 out of 5 gonopores were then selected and secured in the OWT, which was already filled with ambient seawater with a velocity and wave period selected. The trial was initiated when the motor and underlying current were re-started. Time from placement of the urchins in the OWT to when the trial was initiated was less than 2 minutes.

Prior to each experiment, a control sample of eggs and sperm were assayed to: 1) determine the absence of abnormal (e.g., immature) eggs, 2) test whether the male and female pair were compatible [26], [27], and 3) ensure that no eggs had raised membranes that might represent either sperm contamination or mechanical damage. In the compatibility test, if less than 95% of the eggs were fertilized for any pair, new animals were selected. The OWT was housed in a marine lab at the University of New England in Biddeford, Maine, and seawater was pumped from an estuary in a region that was essentially devoid (due to past harvesting) of adult sea urchins. Nevertheless, to ensure there were no background sperm in the seawater, control trials with no male urchin were conducted intermittently during the experimental period (*n* = 5).

Fertilization was assessed as a time integrated measure during a 1 h period [5]. Eggs were sampled at 5 time points (0, 16, 28, 40, and 60 min), and trials were terminated when either 5 samples had been collected or the male or female gamete piles were exhausted. At each time point, eggs were sampled from the water column, wake eddy, substrate and aboral surface respectively. All samples were collected on the downstream side of the female in relation to the underlying current except for the aboral surface sample. For the water column, a 1 L sample was siphoned 0.7 m downstream of the female 0.1 m above the substrate. The wake eddy was sampled with a 50 mL syringe with a narrow extension tube to ensure minimal disruption to the wake eddy 0.05 m downstream of the female. Both the substrate and the aboral surface were carefully sampled with a long 5 mL Pasteur pipette. The substrate was sampled immediately beside the female. Samples from each location were immediately filtered through a 45 *μ*m mesh sieve and the eggs were rinsed with aged seawater (24 – 48 h to eliminate ambient sperm) into separate 20 mL scintillation vials containing a mixture of 2 mL 37% formaldehyde and 10 mL aged seawater within two minutes of collection. During each trial, observations were also recorded of the female and male spawning.

The percentage of eggs fertilized (PF) for each sample was calculated from a random sub-sample of at least 100 eggs or from all the eggs collected in a sample if the number of eggs collected was <100, but >10. Samples with less than 10 eggs were not included in the analysis.

### Effect of hydrodynamics on fertilization

To determine how water motion influences the percentage of eggs fertilized (PF) at each location (water column, wake eddy, substrate, aboral surface) we calculated a mean, weighted by sampling time, at each of 5 sample periods. These numbers represent the percentage of eggs found at a location that were fertilized and do not account for the number of eggs at each location. To examine how different components of flow affect PF we used a stepwise regression (IBM SPSS Statistics V.19) of arcsine-transformed values of PF against the components of flow (current (*U*), wave component (rms(*u_w_*)), turbulent component (rms(*u_t_*)) and period (*T*)). We also conducted these regressions using the turbulent kinetic energy dissipation rate (*ε*), a potentially better predictor of water column processes, instead of rms(*u_t_*). We then compared the resultant regressions to those obtained with the total flow signal (rms(*u*)) and *KC* alone to (i) determine whether using the easily measured rms(*u*) impacts our ability to predict fertilization and (ii) determine if *KC*, a dimensionless parameter that incorporates the period would improve our predictions of fertilization. Before running the analyses all parameters were tested for normality. If the parameters were found to be non-normally distributed they were normalized using a square root transformation. Current (*U*) and the wave component of flow (rms(*u_w_* )) were highly collinear. Rather than using both of these terms in the model, we regressed current versus the wave component and used the residuals of the regression in the model, thus we are asking whether aspects of the underlying current, in addition to those that are directly collinear with the wave component, have effects on fertilization. Our intent was to keep current constant in these experiments thus it did not vary a great deal, additionally the OWT design did not allow for high currents, thus we are simply testing the impacts of small variations in current on fertilization.

Since the percent of eggs fertilized does not provide information about the relative contribution each location makes to total fertilization, we calculated the percentage of the total number of eggs spawned that were fertilized (PFT  =  [PF * # of eggs in a location]/total # of eggs spawned) and the relative contribution of each location to the total number of eggs fertilized (RCO). Eggs did not accumulate on the substratum and were not traceable in the eddy. Therefore, we only estimated the relative contribution of the aboral surface and water column.

To perform these calculations we required an estimate of the total number of eggs spawned by an average female *S. droebachiensis*. Six replicate females were allowed to spawn for one hour in flowing seawater at a low flow rate of 0.02 m s^−1^. At this flow speed, large egg piles form on the aboral surface of female sea urchins and loss of eggs by advection is low [5]. After one hour, the egg piles were gently siphoned off each female’s aboral surface and counted. The mean number of eggs spawned by the six replicate females was 2.2×10^7^, SD  =  4.5×10^5^. This number may be an underestimate of the total number of eggs because of some loss by advection during the hour; however at this low flow loss was minimal.

To establish a nondestructive method of estimating the numbers of eggs in the piles retained on the aboral surface of the urchins at the end of each trial, we established a relationship between the number of eggs in the piles and the volume of the piles. For each individual spawned (see above), we photographed the piles from the top and side. The volumes of piles were estimated from the photographs using Image-Pro® Plus (Media Cybernetics, version 4.1 for Windows™), based on equations for the closest simple geometric shapes (i.e., cones and ellipses) effectively providing a 3D representation of the egg piles. We then compared these estimated volumes to the numbers of eggs that were counted in each pile. Regressions of number of eggs enumerated versus volume of piles estimated from photographs yielded good approximations (Aboral  =  13×10^4^ eggs mL^−1^, *R* ^2^  = 0.92, *n* = 6). For any egg piles that did remain on the surface of the female urchin at the end of a trial we could thus use the regression equation and photographs to quantify the number of eggs. The eggs remaining on the female were then subtracted from the total number of eggs that a female urchin could produce to obtain an estimate of the number of eggs transported unfertilized into the water column (total number spawned – number left at end  =  total number of eggs transported off of the aboral surface).

We assumed that the remainder of the eggs that were not fertilized on the aboral surface passed into the water column and determined the number of these eggs fertilized based on fertilization success found in the water column. Our assumptions, however, overestimate the contribution of the water column to fertilization, especially at low flows, as we know that the substrate and wake eddy can be important sites for fertilization [5]. For each location we then calculated the mean and standard error of the number of eggs that were fertilized in three rms(*u*_w_) categories: LOW [<0.03 m s^−1^ (*n* = 10)], MEDIUM [>0.03<0.06 m s^−1^ (*n* = 7)], HIGH [>0.06 m s^−1^ (*n* = 5)].

## Results

As noted in earlier investigations [5], visual observations at the start of each experiment confirmed the accumulation of eggs and sperm in piles on the aboral surface of the spawning sea urchins. Our observations suggested that the speed at which the piles were depleted was dependent on the velocity and period of the experimental flow conditions, with higher rms(*u*_w_) values associated with increased rates of gamete erosion. Irrespective of the presence of piles of eggs and sperm on the parent surface, in all experimental trials both male and female animals were observed to continue spawning throughout the duration of the experimental period of 1 hour.

These visual observations also provided general information on the movement of eggs around female animals under the various experimental conditions. Over the range of flow conditions generated, eggs were consistently entrained from the aboral surface of the female into the vortex that formed downstream of the body of the animal. Immediately following reversal of the flow in the oscillating flow-field, the vortex structure broke down and the eggs were carried back over the animal and became entrained in the newly formed vortex on the opposite side of the animal. The transfer of eggs from one side of the animal to the other also resulted in some transport of the eggs into the overlying water column. We also observed that except for the lowest rms(*u*_w_) <0.01 m s^−1^, no piles of eggs were formed directly beside the female, although even at the lowest rms(*u*_w_) values eggs could be seen rolling along the substrate (essentially transported as bed load).

**Figure 4:**
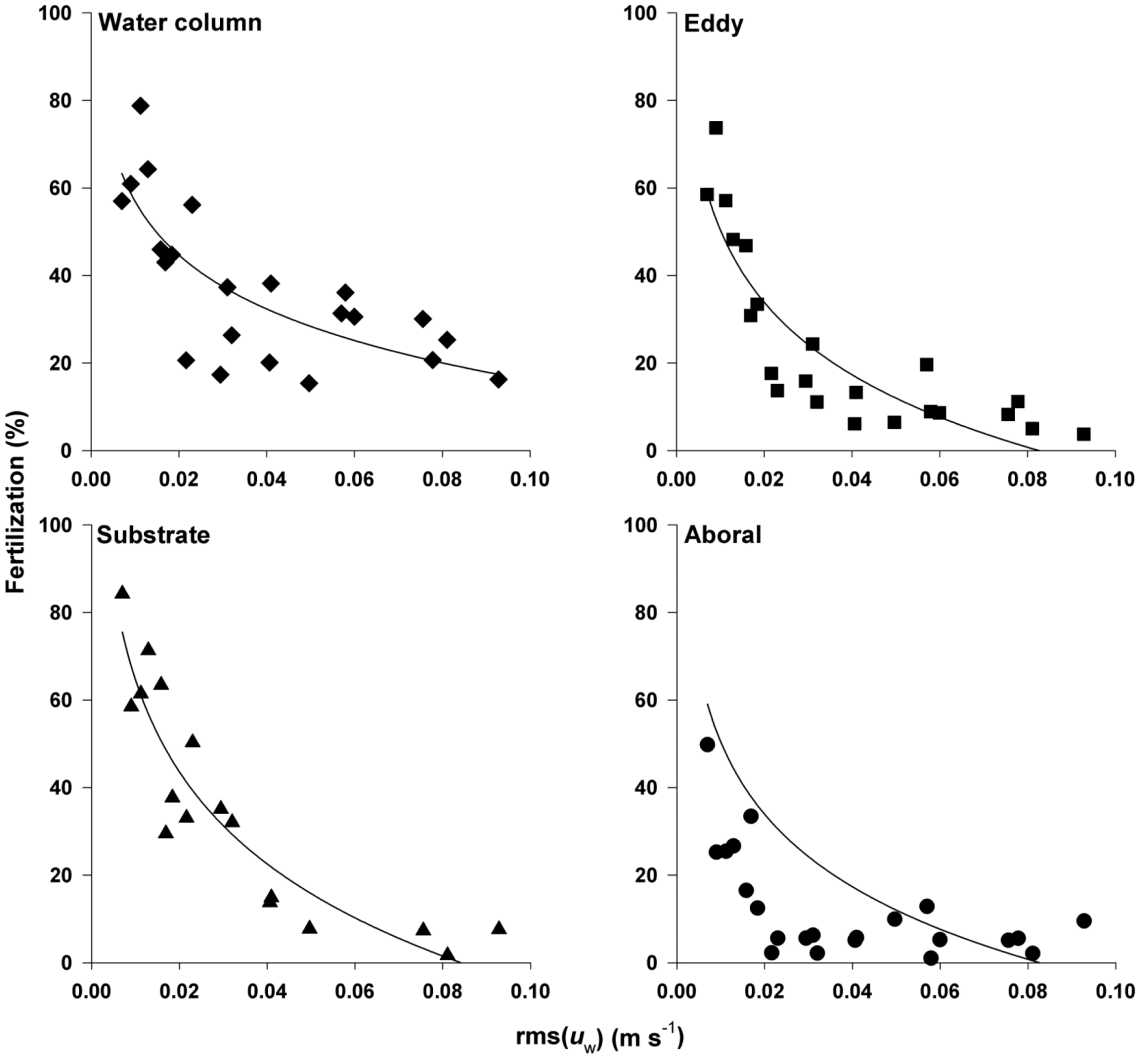
Variation in fertilization as a function of flow. Mean percent fertilization (PF) is plotted as a function of rms(*u*_w_) (m s^−1^) in the four sampling locations: water column, wake eddy, substrate, and aboral surface. Symbols represent weighted mean of all the time points. Lines represent a nonlinear curve fit to the untransformed data. PF was strongly dependent on rms(*u*_w_) (m s^−1^) for all sampling locations (Table 1).

At low flows (rms(*u_w_*) <0.02 m s^−1^ ) for the majority of the sampling locations (water column, wake eddy and substrate), PF were high with 60 – 85% of the eggs fertilized (Fig. 4). The exception was the aboral surface where maximum PF was only ∼50%. As rms(*u_w_*) increased, fertilization decreased to below 10% for all sampling locations except the water column where fertilization remained at 20% (Fig. 4).

A stepwise regression of the components of flow (current (*U*), wave component (rms(*u_w_*)), turbulence component (rms(*u_t_*)) and period (*T*)) indicated that for all the sampling locations except the substrate only rms(*u_w_*) had a significant relationship to the percentage of eggs found fertilized at each location (Table 1). For the substrate, including period in the regression improved model fit. Interestingly, including the turbulence component (rms(*u_t_*)) or energy dissipation (*ε*) in the models did not improve the fit. Thus larger scale measures of flow that are correlated with smaller scale fluctuations are more predictive of fertilization under these flow conditions than measures of small scale turbulent mixing. A comparison of the stepwise regressions fits of rms(*u_w_*) and those using rms(*u*) and *KC* alone as predictors of fertilization indicated that the stepwise regressions provided slightly better fits than either rms(*u*) or *KC* at most sampling locations (Table 1 & 2). Using rms(*u*) does provide significant predictions, however, and is more easily calculated than the separate components.

**Table 1:** Stepwise regression analyses.

Location	Step	*r* ^2^	Delta *r* ^2^	*T*	*p*
Water column	1. Waves	57.8	57.8	–5.24	0.0001
Eddy	1. Waves	81.8	81.8	–9.48	0.0001
Substrate	1. Waves	77.7	77.7	–8.35	0.0001
	2. Period	84.6	6.9	–2.91	0.009
Aboral	1. Waves	55.2	55.2	–4.96	0.0001

Results of stepwise regression analysis for the four components of flow: current (*U* m s^−1^), wave (rms(*u_w_*) m s^−1^), turbulent (rms(*u_t_*) m s^−1^), and period (*T* (s)), vs. arcsine-transformed values of PF at the four sampling locations. Stepwise regressions were done with *p*<0.05 to enter and *p*<0.10 to remain.

**Table 2:** Regression analyses for percent eggs fertilized (PF).

Location	rms(*u*) (m s^−1^)	*KC*
Water column	55.1 (*F*_1,20_ = 24.56)	49.5 (*F*_1,20_ = 19.66)
Eddy	76.8 (*F*_1,20_ = 66.0)	64.0 (*F*_1,20_ = 35.64)
Substrate	84.0 (*F*_1,15_ = 78.99)	88.3 (*F*_1,15_ = 113.53)
Aboral	49.2 (*F*_1,20_ = 19.38)	61.5 (*F*_1,20_ = 31.94)

Results of regression analysis for the two flow parameters: Total flow signal (rms(*u*)) and *KC*, vs. arcsine-transformed values of PF at the four sampling locations. Values are *r* ^2^ with df and *F*-ratio in parentheses. All predictors were significant to the 0.0001 level.

**Figure 5:**
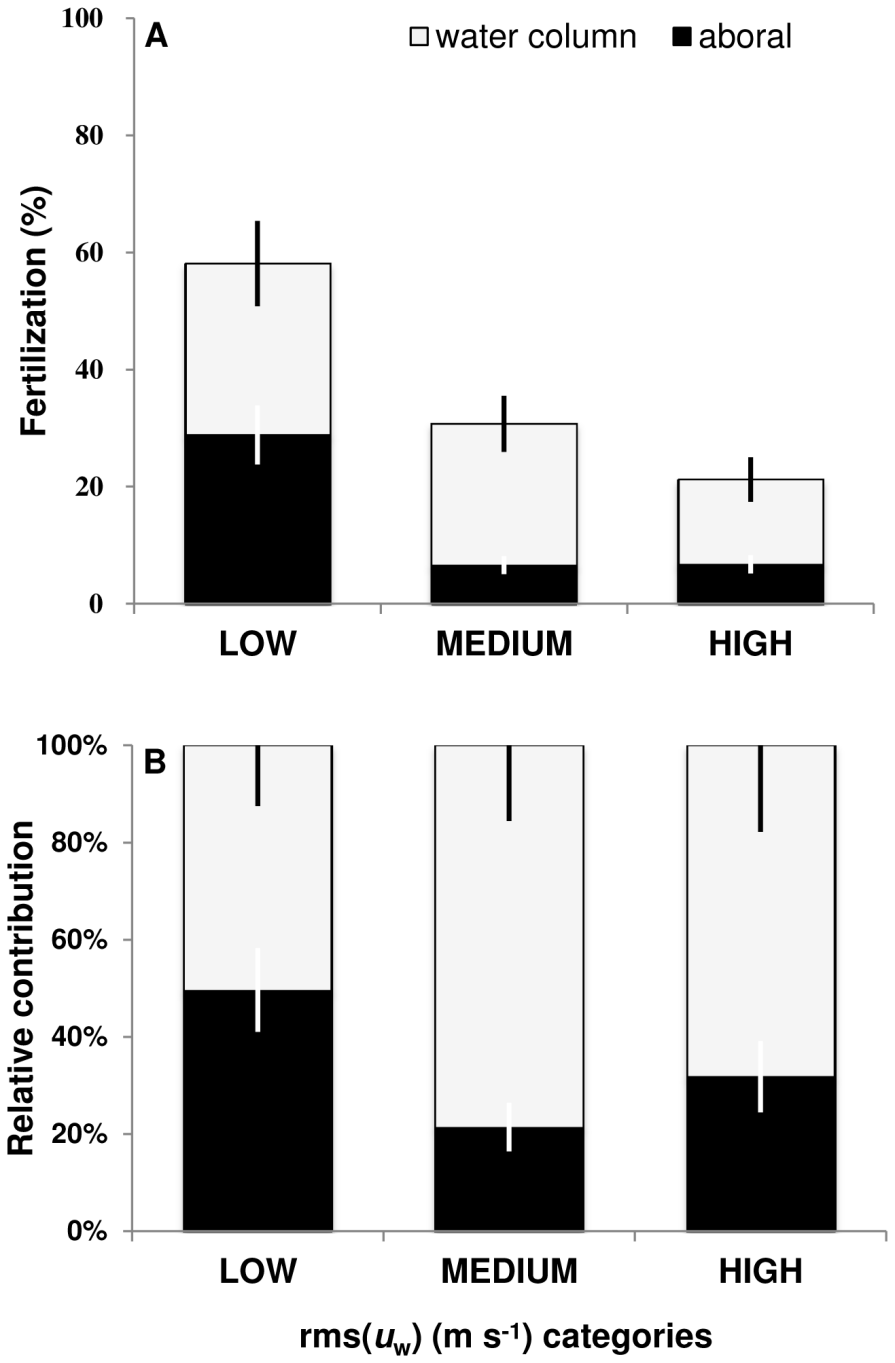
Importance of location for egg fertilization. Estimate of (A) the percentage of the total number of eggs spawned that were fertilized (PTF) and (B) the relative contribution to overall fertilization (RCO) at the aboral surface and water column as a function of rms(*u*_w_) categories: LOW (<0.03 m s^−1^), MEDIUM (>0.03<0.06 m s^−1^) and HIGH (>0.06 m s^−1^).

The percentage of total number of eggs fertilized (PFT) on the aboral surface and water column samples declined between low and medium rms(*u_w_*) categories and remained constant between the medium and high rms(*u_w_*) categories (Fig. 5A). Similar PFT were observed between the aboral surface and water column (∼30%) at low flows, however as rms(*u_w_*) categories increased, only 6% of the eggs on the aboral surface were fertilized compared to ∼ 25% in the water column (Fig. 5A). The relative contribution (RCO) was similar between the aboral surface and water column at low rms(*u_w_*) categories. At medium and high categories, fertilization was lower at the aboral surface than in the water column with approximately 25% of the eggs being fertilized compared to ∼75% for the water column (Fig. 5B).

Controls to determine background sperm levels in the OWT indicated that no significant sperm concentrations were present within the OWT with >98% of the eggs remaining unfertilized.

## Discussion

The root-mean-square of the total flow signal (rms(*u*)) was a relatively good predictor of fertilization at all sampling locations around a spawning female (p<0001). This parameter is composed of the underlying currents (*U*), wave generated fluctuations (rms(*u*_w_)), and turbulent fluctuations (rms(*u*_t_)). In our analyses the wave component rms(*u*_w_) provided a better fit to our data than did rms(*u*) (Table 2). These results indicate that rms(*u*_w_) captures most of the variance associated with processes driving fertilization under the flow conditions tested in our OWT. The small effects attributed by period for the substrate locations are intriguing and suggest that period plays some role in controlling fertilization processes and could suggest that more complex experiments with varying period could be informative. Regardless, from a practical perspective, rms(*u*_w_) can be used as a predictor of fertilization in complex flows and captures the important components of flow.

Due to the potential influence of period on fertilization we compared fertilization success to a non-dimensional parameter *KC*. This parameter represents the ratio of orbital excursion length (length of flow movement in one direction) to animal size. Thus it may be an important concept for analyzing fertilization for a range of animals of different sizes experiencing a range of flow excursion lengths. In our study, using *KC* did not improve our predictions of fertilization, but this may be due to minimal size variation in our urchins. We included *KC* in our analysis, however, as it is a good predictor of fertilization and may prove useful for studies where animals exhibit greater variation relative to excursion length.

Similar to previous studies assessing fertilization in unidirectional flow conditions, fertilization declined with increasing flow speeds [7]-[9]. The percentage of eggs fertilized at low flows (rms(*u*_w_) <0.02 m s^−1^) varied between 50 – 85%, depending on the location sampled (water column, wake eddy, substrate or aboral surface), but declined to levels below 10% for most locations as rms(*u*_w_) increased. Water column fertilization remained at 20% even at these higher flow velocities.

Despite continual fluctuations in flow velocity around the female sea urchin, fertilization occurred in various localities on or near the female. These results extend previous work of Yund and Meidel [5] showing that fertilization is not just a water column process in unidirectional flows. The lowest levels of fertilization occurred on the aboral surface where PF reached a maximum of 50% and was reduced to <10% at rms(*u*_w_) >0.04 m s^−1^. Nonetheless these results indicate that eggs can already be fertilized by the time they leave the female. Eggs leaving the aboral surface unfertilized may still be fertilized for some time, though this concept has received little attention in experimental design [5]. Eggs can become entrained in the vortices on either side of the sea urchin in the oscillating flow-field or transferred to the substrate by the pressure forces acting on the parent. Fertilization can occur in these locations as evidenced by higher fertilization levels in the substrate and wake eddy samples (Fig. 4).

Our results indicate that fertilization can remain upwards of 20% even at relatively fast flows and short periods of oscillation. Furthermore, our results indicate that the water column is an important location for fertilization in oscillatory flow. The total number of eggs fertilized and the relative contribution of the water column is greater than the aboral surface, especially at medium and high rms(*u*_w_) categories. It is important to note, however, that fertilization can and does occur at the aboral surface location and may be a significant contributor to overall fertilization success. Oscillatory flow suspended the eggs in the overlying water column and inhibited the formation of discernible piles of gametes on the substrate. Although we were not able to determine the contribution of fertilized eggs from the wake eddy and substrate to the water column sample, the eggs found in the water column are likely to include eggs already fertilized from all three locations near or on the parent (aboral surface, substrate and wake eddy). Because of the additional contribution of these locations to water column fertilization estimates we may be overestimating the water column contribution. Nevertheless, sea urchin gametes can be successfully fertilized when spawning under a range of oscillatory flow conditions.

Evaluating different hydrodynamic parameters for relating fluid dynamics to fertilization processes is an essential step in establishing possible ecological effects on fertilization [28], [29]. Because of its size, our oscillatory OWT was unable to properly mimic boundary layer effects. The thickness of a boundary layer grows as flow moves over a surface, and the relatively short length of our OWT probably produced boundary layers that were thinner (at the sites of the spawning sea urchins) than in nature. A further consideration of the OWT was that we captured large scale oscillatory processes over a time integrated measure rather than the small scale processes at the scale of fertilization. Shear stress is an important process influencing fertilization at the scale of sperm encountering eggs [30]–[32], and it is possible that the OWT did not correctly capture these small scale processes. Nevertheless we expect that our understanding of fertilization processes should be correct for the rms(*u*_w_) values actually measured in the OWT, but recognize that in the field, similar rms(*u*_w_) values at the sea urchin height above the substrate may occur with different combinations of current and turbulence. Consequently, further work is necessary to establish how parameters measured in our OWT correspond to real-world wave forces. We also held the unidirectional current constant. In nature underlying currents may vary a great deal. Our results indicate that currents contribute to the fertilization occurring within eddies near the spawning female thus underlying currents may influence the relative importance of different locations to fertilization in natural systems.

Our experiments only address the simple spawning scenario of a single male and female (female positioned directly downstream of the male) and therefore are not representative of population-wide spawning events. *Strongylocentrotus droebachiensis* can be found in densities from 0.1 to 250 urchins m^−2^ [9] and are expected to aggregate before a mass synchronous spawning event [6], [33], [34]. Aggregation behavior has been observed in this species and other mobile invertebrates [35]–[37] and can only increase fertilization rates and mitigate the effects of sperm limitation. When individuals are densely packed in oscillatory flow, eggs and sperm may pass over neighbors to the front and back of the spawning individual several times depending on the period and underlying current as observed by [36]. Oscillatory flow may therefore enhance gamete coalescence and increase the number of eggs fertilized in mass spawns relative to unidirectional flow under similar density conditions.

The nature of flow is a fundamental component influencing the success of fertilization and some form of water motion is required to bring sperm into contact with eggs, but there appears to be a fine balance between excessive and sufficient. We know that small scale turbulent processes can result in regions of high concentration of sperm and eggs thereby enhancing fertilization [2], however, turbulence did not explain the variance in fertilization beyond that captured by rms(*u*).

Whilst there are numerous investigations into the cues that trigger large-scale broadcast spawning events [38], [39] and biological conditions, e.g., density and number of spawning individuals [7], [9], [40], we know little about the flow conditions during natural spawning occasions [41]. Sea urchins have been observed to spawn when water flow rates were minimal [34] or in weak to moderate currents [36]. Other taxa such as the anemone *Oulactis mucosa* or fucoid algae have also been observed to either release spores or spawn in the intertidal at low velocities (< 0.2 m s^−1^) or at low tide in rock pools [42]–[44]. Quantitative hydrodynamic information during natural spawning events for subtidal species is therefore essential if we are to understand and model fertilization success in the environment. Based on our experience the most adaptive strategy would be for urchins to synchronize spawning events during low flow conditions.

In summary, although we perceive the wave environment to be extremely turbulent and therefore expect fertilization levels to be low, our expectations are not always valid. Our results demonstrate that under certain oscillatory flows, the percentage of eggs fertilized can attain high levels (50–80%). These results highlight the fact that gametes can attain successful fertilization in energetic coastal wave environments. In addition, fertilization can occur on the aboral surface before eggs mix into the water column and fertilization rates can be quite high depending on flow conditions.
